# Supplementary material for: An Epstein‐Barr Virus Nuclear Antigen 1 (EBNA1) Serology Test Strip for Nasopharyngeal Carcinoma Risk Screening
Source: J Med Virol. 2026 Jul 27;98(8):e71082. doi: 10.1002/jmv.71082 (PMC13402912; doi:10.1002/jmv.71082)
Supplement: Supplementary file 1 — Supporting File [file JMV-98-e71082-s001.pdf]

Supplementary Materials for

An Epstein-Barr virus nuclear antigen 1 (EBNA1) serology test strip for nasopharyngeal carcinoma risk screening

Benjamin E Warner[1,2,3], Japan Patel[1,4], Rebecca Satterwhite[1,3,5], Renwei Wang[5], Jennifer Adams-Haduch[5], Woon-Puay Koh[6], Jian-Min Yuan[3,5†*], Kathy H Y Shair[1,5,7†*]

*Corresponding author. Email: kas361@pitt.edu

This supplementary file includes:

Supplementary Tables 1-2
Supplementary Figures 1-8

SUPPLEMENTARY TABLES

Supplementary Table 1. Baseline demographic and lifestyle characteristics of study participants who developed nasopharyngeal carcinoma (cases) and healthy subjects (controls) from the Singapore Chinse Health Study and Shanghai Cohort Study.

Characteristic	Singapore Chinese Health Study			Shanghai Cohort Study		
	Cases	Controls	*p*[a]	Cases	Controls	*p*[a]
Number of subjects (incident NPC)	13	61		7	35	
Time to diagnosis (year), median (range)	1.8 (0.4-3.9)	n/a		1.3 (0.1-3.1)	n/a	
Follow up (year), median (range)	n/a	12.9 (5.5-19.8)		n/a	28.1 (4.4-31.6)	
Age (year), mean ± SD	58.2 ± 6.6	59.2 ± 5.9	0.581	55.3 ± 3.0	55.0 ± 3.2	0.822
Body mass index (kg/m^2)	22.5 ± 2.4	23.3 ± 3.5	0.401	23.0 ± 5.1	22.3 ± 3.5	0.654
Highest level of education, %						
No formal education	7.7	4.9		0	5.7	
Primary school	46.2	44.3		14.3	31.4	
Secondary school or above	46.2	50.8	0.901	85.7	62.9	0.483
Cigarette smoking, %						
Never smokers	53.9	57.4		42.9	62.9	
Former smokers	0	21.3		0	2.8	
Current smokers	46.1	21.3	0.068	57.1	34.3	0.499
Alcohol drinking, %						
Nondrinkers	69.2	63.9		57.1	62.9	
Moderate drinkers	23.1	36.1		28.6	34.3	
Heavy drinkers[b]	7.7	0	0.072	14.3	2.8	0.430

[a]2-sided *p* values were based on *t* test for continuous variables or chi-square test for categorical variables; [b]Heavy drinking was defined as >3 drinks/day for men and >2 drinks/day for women, and lower levels as moderate drinking

Supplementary Table 2. EBNA1 genotype of pre-diagnostic sera that tested negative by EBNA1 SeroStrip-HT.

		EBNA1 Serotype		EBNA1 Genotype	
Cohort	Years pre-diagnosis	Prototype Assay[19] *mamEBNA1 dGAr*[a]	EBNA1 SeroStrip-HT *mamEBNA1 dGAr*[b]	Total Reads	% Reads by Residue 487
SCS	0.06	Positive	Negative	143,372	99% 487V<br>1% 487A
SCHS	1.80	Positive	Negative	137,091	97% 487V<br>3% 487A
SCS	2.07	Positive	Negative	ND	ND

SCS; Shanghai Cohort Study, SCHS; Singapore Chinese Health Study, ND; not detected, 487V or A; EBNA1 residue at position 487 relative to EBV GenBank ref. V01555.2

[a]Purified EBNA1 dGAr from mammalian cells, [b]Whole-cell lysate from mammalian cells containing EBNA1 dGAr

SUPPLEMENTARY FIGURES

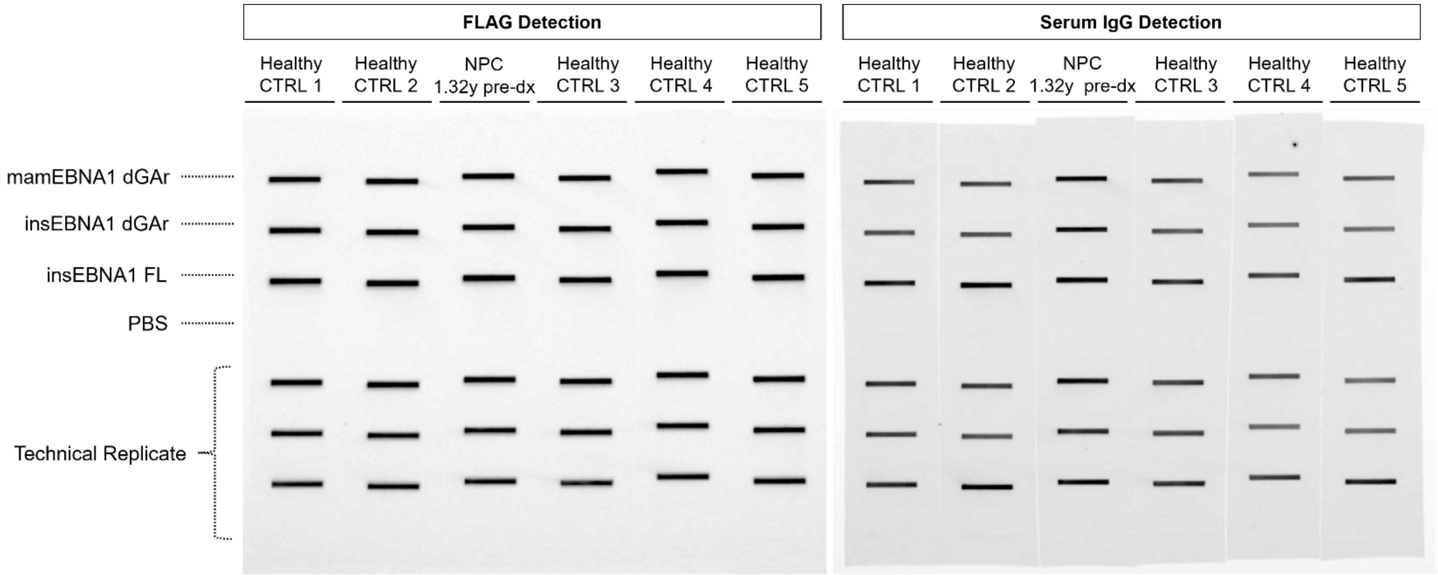

Supplementary Figure 1. EBNA1 SeroStrip-HT example of FLAG channel participant EBNA1 IgG detection. Protein analytes were loaded in duplicate onto nitrocellulose including EBNA1 deleted for the glycine-alanine repeat produced in mammalian cells (mamEBNA1 dGAr) or insect cells (insEBNA1 dGAr), and full-length EBNA1 produced in insect cells (insEBNA1 FL). Phosphate-buffered saline (PBS) was included as a background control. An N-terminal FLAG-tag permitted fluorescent visualization of each analyte. The IgG channel of the pre-diagnostic (pre-dx) NPC sera 1.32 years to diagnosis is displayed with 5 matched healthy control subjects (see Figure 1 for IgA detection). Densitometry was used to quantify participant antibody detection intensity of each analyte.

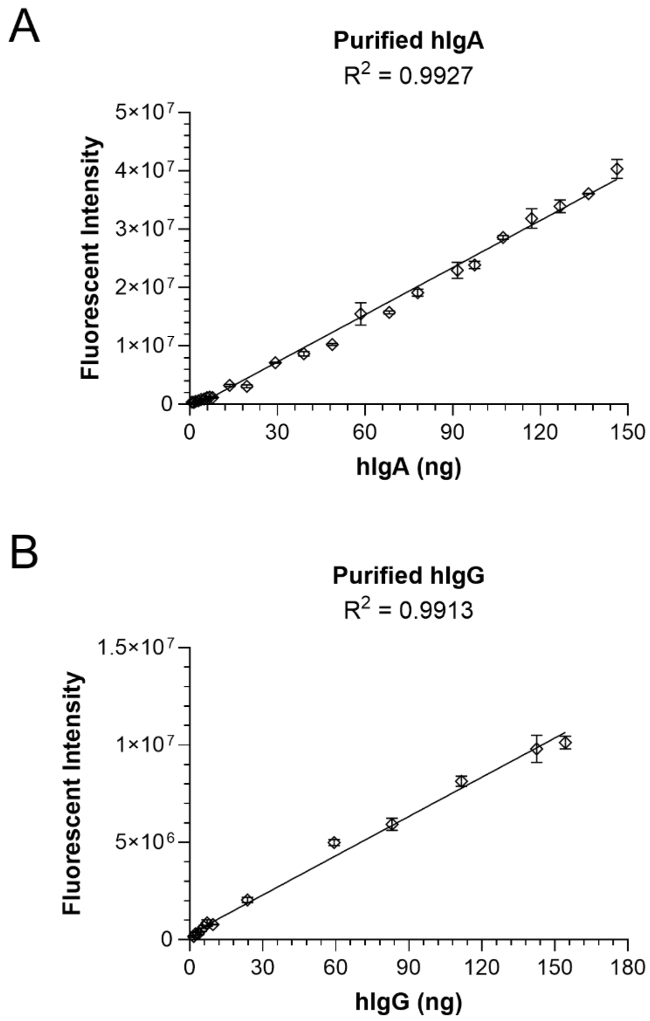

Supplementary Figure 2. Standard curves for purified human IgA and IgG.

(A) Known quantities of purified human IgA monomers were loaded onto a nitrocellulose membrane, fluorescently stained, and measured by densitometry to produce a standard curve for absolute quantification of EBNA1 IgA. The same was done for **(B)** purified human IgG monomers to generate a standard curve. R^2; goodness of fit.

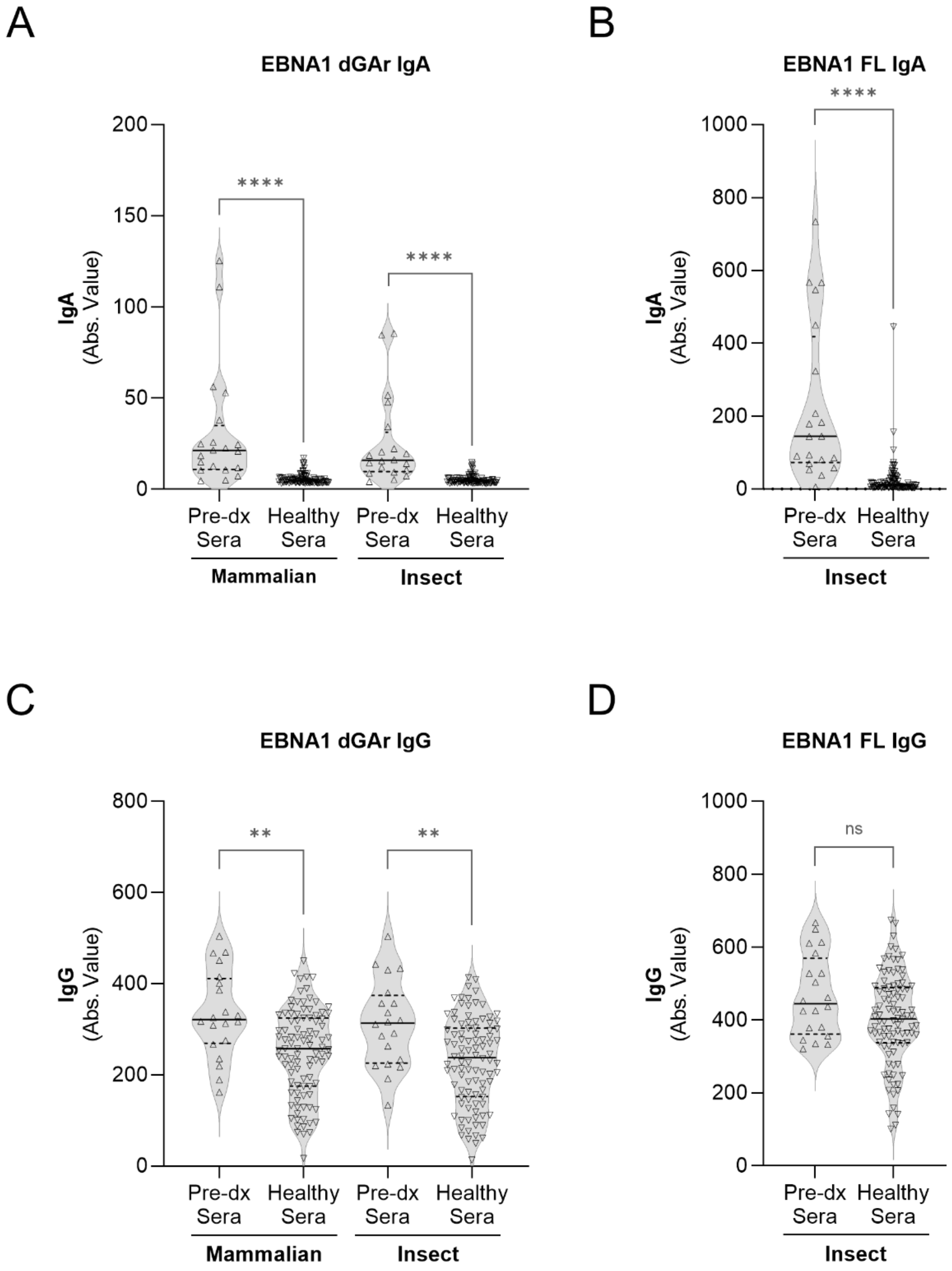

Supplementary Figure 3. Absolute quantification of EBNA1 SeroStrip-HT IgA and IgG detection across pre-diagnostic and healthy sera.

Pre-diagnostic sera (n=20) and healthy (n=96) sera were measured for **(A)** IgA against EBNA1 deleted for the glycine-alanine repeat produced in mammalian cells (mamEBNA1 dGAr) or insect cells (insEBNA1 dGAr), **(B)** IgA against full-length EBNA1 produced in insect cells (insEBNA1 FL), **(C)** IgG against mamEBNA1 dGAr or insEBNA1 dGAr, **(D)** IgG against insEBNA1 FL. Within violin plot, Solid horizontal bar; median, Dotted horizontal bar; upper and lower quartiles. ****; *P*<0.0001, **; *P* <0.005, ns; not significant.

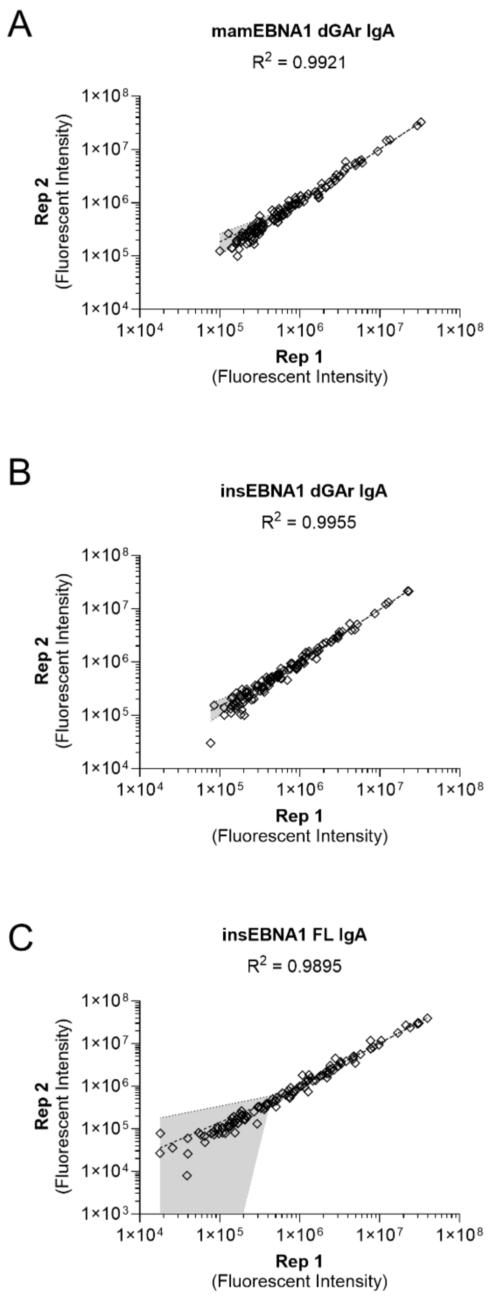

Supplementary Figure 4. Correlation of Replicate 1 and 2 IgA detection for each EBNA1 analyte. The fluorescent intensity of Replicate 1 (x-axis) compared to Replicate 2 (y-axis) IgA detection of (**A**) mammalian-derived EBNA1 deleted for the glycine-alanine repeat (mamEBNA1 dGAr), (**B**) insect-derived EBNA1 deleted for the glycine-alanine repeat (insEBNA1 dGAr), and (**C**) insect-derived full-length EBNA1 (insEBNA1 FL). R^2; goodness of fit, Shaded region; 95% confidence band.

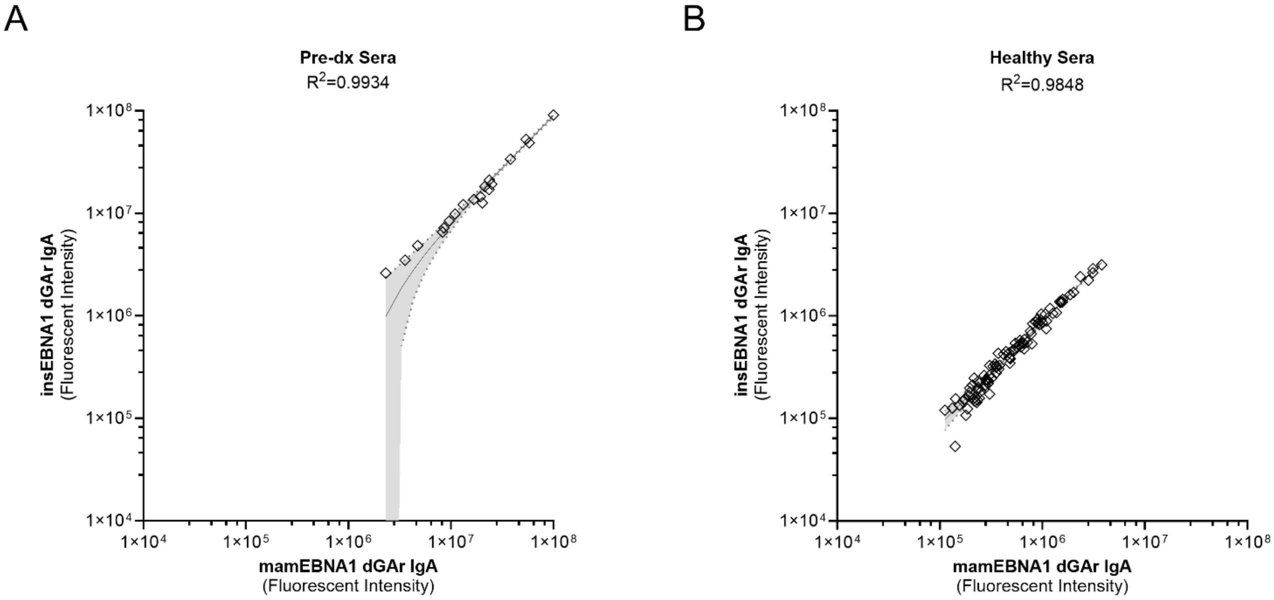

Supplementary Figure 5. EBNA1 IgA detection correlation between mammalian- and insect-derived proteins. The fluorescent intensity of IgA detection of mammalian-derived EBNA1 dGAr (x-axis) compared to insect-derived EBNA1 dGAr (y-axis) in pre-diagnostic sera (left) and healthy sera (right). R2; goodness of fit, Shaded region; 95% confidence band.

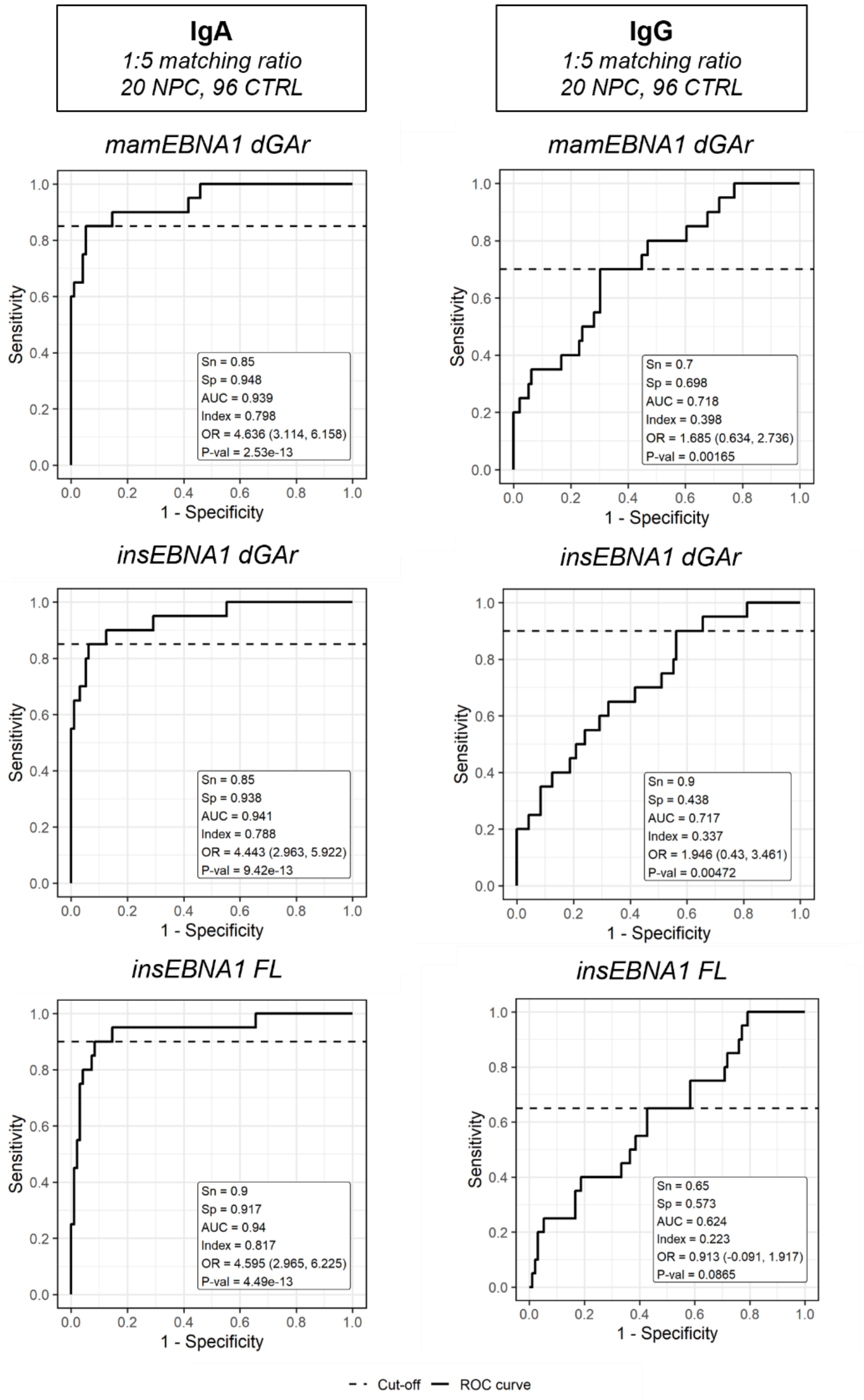

Supplementary Figure 6. Receiver Operating Characteristic curves for EBNA1 analytes in a 1:5 case-control matching ratio.

Receiver operating characteristic curves were generated for IgA (left) and IgG (right) detection of EBNA1 analytes from 20 pre-diagnostic sera and 96 healthy control sera to identify cutoffs for optimal sensitivity and specificity determined by maximal Youden's index. Cutoff (abs. value), mamEBNA1 dGAr IgA; 10.47, insEBNA1 dGAr IgA; 9.29, insEBNA1 FL; 51.96, mamEBNA1 dGAr IgG; 307.60, insEBNA1 dGAr IgG; 215.60, insEBNA1 FL; 424.30.

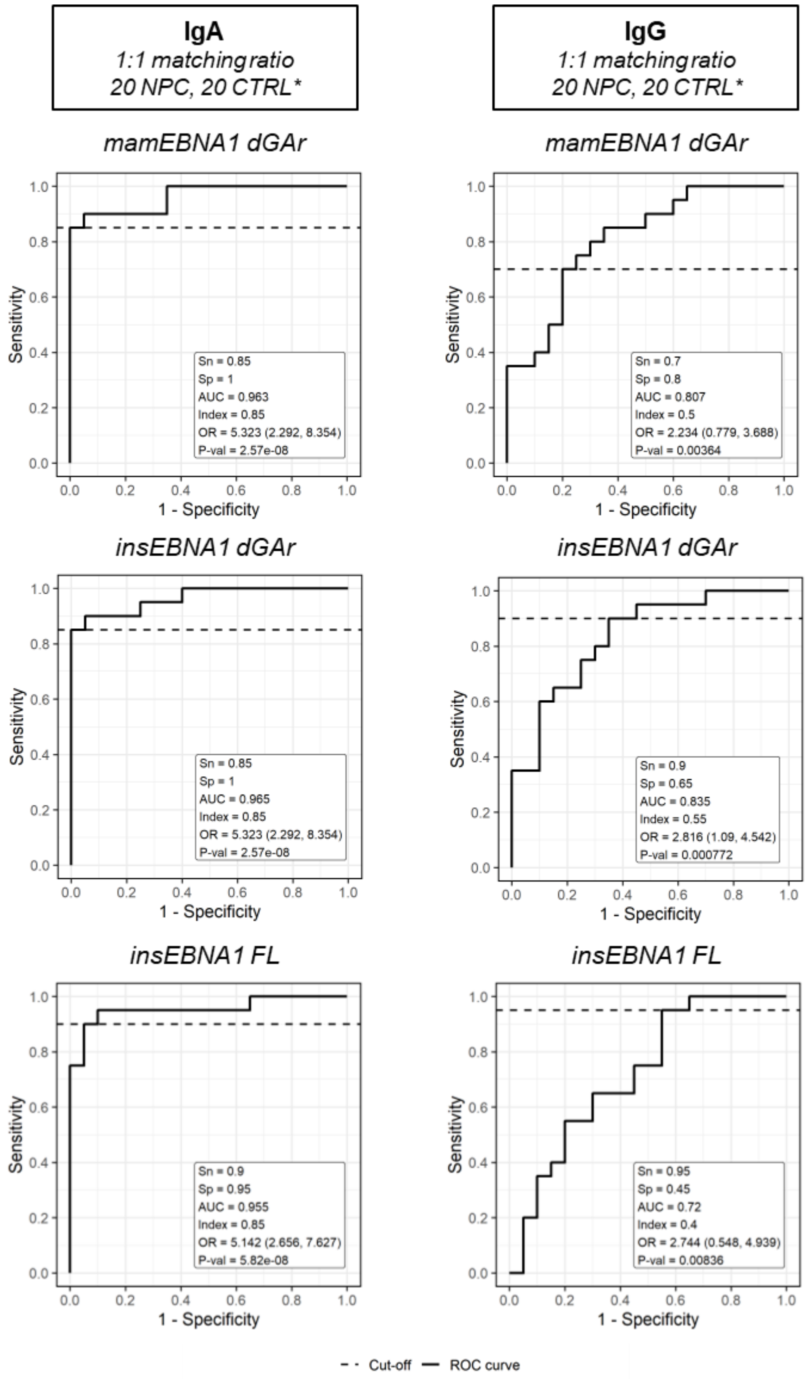

Supplementary Figure 7. Receiver Operating Characteristic Curves (ROCs) for EBNA1 analytes in a 1:1 case-control matching ratio.

Receiver operating characteristic curves were generated for IgA (left) and IgG (right) detection of EBNA1 analytes from 20 pre-diagnostic sera and 96 healthy control sera to identify cutoffs for optimal sensitivity and specificity determined by maximal Youden's index. Cutoff (abs. value), mamEBNA1 dGAr IgA; 9.60, insEBNA1 dGAr IgA; 8.42, insEBNA1 FL; 50.67, mamEBNA1 dGAr IgG; 307.60, insEBNA1 dGAr IgG; 214.90, insEBNA1 FL; 331.00. *1:1 case-control matching preserved from Warner *et al.* Clin Can Res (2024), ref.19.

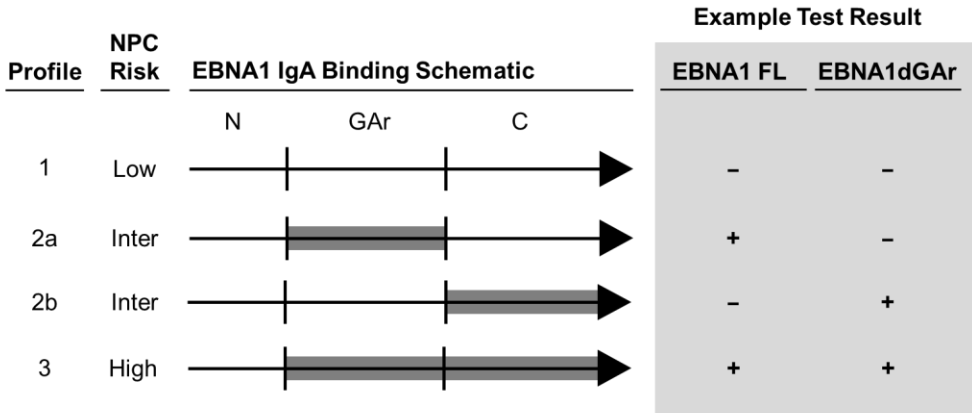

Supplementary Figure 8. NPC risk profiles based on IgA detection of specific EBNA1 epitopes. Schematic EBNA1 epitope recognition by IgA and risk of NPC diagnosis. Four profiles were established based on IgA against mamEBNA1 dGAr and insEBNA1 FL in a simultaneous testing model. Example test result shown with positive (+) indicating value above cutoff and negative (-) indicating below cutoff. Cutoff (abs. value) from 1:5 matching ratio analysis; mamEBNA1 dGAr; 10.47, insEBNA1 FL; 51.96.
